# Nutritional Interventions for Patients with Severe Obesity Seeking Bariatric Surgery

**DOI:** 10.3390/nu15030515

**Published:** 2023-01-19

**Authors:** Tair Ben-Porat, Shiri Sherf-Dagan

**Affiliations:** 1 Department of Health, Kinesiology, and Applied Physiology, Concordia University, Montréal, QC H4B 1R6, Canada; 2 Montreal Behavioural Medicine Centre (MBMC), Centre Intégré Universitaire de Santé et de Services Sociaux du Nord-de-l’Île-de-Montréal (CIUSSS-NIM), Montréal, QC H4J 1C5, Canada; 3 Department of Nutrition Sciences, School of Health Sciences, Ariel University, Ariel 40700, Israel; 4 Department of Nutrition, Assuta Medical Center, Tel Aviv 69710, Israel

Bariatric surgery (BS) is usually considered when other weight-loss treatments have failed, and remains an effective long-term treatment for severe obesity and its related medical complications [1]. However, a substantial proportion of individuals who undergo BS achieve suboptimal outcomes, in terms of weight loss, remission of medical-related complications, development of maladaptive eating behaviors, poor psychological adjustment, and nutritional deterioration and its related longer-term metabolic complications [2,3]. As a result, a subset of patients may require escalation of therapy, a new obesity treatment modality, or even revisional BS [4]. Suboptimal postoperative weight results are often attributed to a return to maladaptive lifestyle behaviors, poor adherence to the postoperative diet, inadequate follow-up support with the dietitian, or low patient knowledge, rather than surgical or metabolic reasons [5,6,7]. Therefore, pre- and postoperative nutritional intervention programs should be studied, and, if successful, be implemented into clinical practice [8,9].

Some groups within the BS population may be at potentially higher nutritional risk. These include, but are not limited to, patients with extreme body mass index (BMI), sarcopenic obesity, multiple nutritional deficiencies, poor glycemic control, poor oral hygiene, advanced kidney disease, untreated eating disorders or maladaptive eating behaviors, lack of nutritional knowledge related to BS, childbearing-age women seeking future pregnancy, and candidates for a revisional BS [8]. Although there is paucity of literature regarding these groups in terms of nutritional assessment, preparation, and outcomes, in clinical practice they are often required to undergo more extensive nutritional assessments and be targeted for specific nutritional intervention during the preoperative period [8]. Here, we address four prominent at-risk groups within the population of patients living with severe obesity who seeks BS. The scientific literature regarding pre-surgical nutritional intervention strategies that aim to prevent postoperative nutritional and metabolic complications and improve postoperative health outcomes is summarized.

***Patients with extreme BMI.*** Severe obesity (i.e., BMI ≥ 50 kg/m^2^) can impose higher surgical technical challenges, higher risk of complications, and a lower chance of achieving primary weight loss goals post BS [10,11]. Therefore, it is reasonable to consider bridging interventions for weight loss before surgery within this population. Proposed interventions include first-step laparoscopic sleeve gastrectomy, intragastric balloon, anti-obesity pharmacotherapy, and liquid low-calorie diet programs (i.e., <800–900 kcal/day) [12,13], with the last having led to a preoperative mean difference of 9.8 kg/m^2^ BMI loss according to a recent published systematic review [10,11]. Collectively, studies on bridging interventions for patients with BMI ≥ 50 kg/m^2^ are very limited and there is still gap of knowledge regarding the long-term effectiveness of BS with or without the use of bridging interventions in this population. Therefore, there are presently no uniform clinical recommendations regarding the optimal preoperative management or dietary treatment of this population [8,10,11]. Nonetheless, besides obesity level, more factors should be taken into consideration when prescribing a perioperative diet, such as patient’s age, the presence of medical-related complications, and behavioral and psychological aspects [14].

***Patients with impaired nutritional status and multiple deficiencies prior to BS.*** Impaired pre-surgery nutritional status is found to be related to postoperative nutritional deficiencies and can be associated with further metabolic complications, such as bone loss [8,15]. Treating nutritional deficiencies after surgery can be complex, especially in light of patients’ limited adherence to clinical care and the supplementation regime during the postoperative period, particularly in the longer-term after the surgery [15]. In regard to routine supplementation regimes at the pre-surgical period, a few recent trials testing vitamin D loading at the pre-surgery period demonstrated some benefits for the pre- and postoperative levels of this vitamin as well as for some skeletal parameters, when combined with other interventions post BS (e.g., calcium supplements and physical activity) [16,17,18]. However, the impact of other supplementation programs targeting specific nutrients (e.g., iron, folate, vitamin B12, etc.) were tested mostly at the postoperative period [19,20]. Most available guidelines and position statements to date emphasize the need to correct preoperative nutritional deficiencies, and some suggest multivitamin supplementation during the preoperative diet period [8,9,21]. Importantly, although micronutrient deficiencies have not been identified as an absolute contraindication to BS, special attention should be paid to patients with a higher risk of multiple nutritional deficiencies, including those with higher class of obesity, candidates for malabsorptive procedures, cases of revisional procedures, and certain ethnic subgroups [22].

***Patients with a poor glycemic control.*** Achieving preoperative optimal glycemic control can reduce the lengths of hospital stay, risk of wound infections, and further postoperative complications [8,23]. The targets for preoperative glycemic control include hemoglobin A1c (HbA1c), with a value of <7.0% in general, but <8.0% in cases of advanced macrovascular or microvascular complications, extensive comorbid conditions, or long-lasting disease [8,23]. A few small-scale studies have found dietary program to improve glycemic control in pre-surgical patients [24,25,26]. One of these studies reported an association between a rapid glycemic response to a preoperative 2-week low-calorie diet (i.e., 1100 calories per day for women and 1300 calories per day for men) and a postoperative early remission of type 2 diabetes mellitus (T2DM) among BS patients with insulin-dependent T2DM [25]. Another study tested an interprofessional glycemic optimization program which included individualized nutritional counseling, exercise prescription, adjustment of antihyperglycemic therapy, and weekly phone calls, among BS candidates with poor glycemic control (i.e., HbA1c values of 9.0% ± 1.2%), and reported that 92% of participants reached the target HbA1c values of ≤8.0% [24]. In a pilot randomized controlled trial (RCT) study among 34 patients with uncontrolled diabetes (i.e., HbA1c ≥ 8.5%) who were randomized to receive either glucose optimization or not for 3 months preoperatively, 30-day perioperative complication, length of stay, and glycemic control at 1 year post-surgery were similar between the groups, although glycemic control was superior among the glucose optimization group post-intervention [26]. Recent international association guidelines state that pre-procedure glycemic control must be optimized using a diabetes comprehensive care plan, including healthy, low-calorie dietary patterns, medical nutrition therapy, physical activity, and pharmacotherapy when necessary [23]. Moreover, preoperative weight loss using a very-low- or low-calorie diet for 2–4 weeks was recommended for patients with T2DM to improve insulin sensitivity, although it is important to remember that the effect of preoperative weight loss regimes within the population of patients with obesity and poor glycemic control has barely been investigated so far [27]. Finally, it is important to mention that any adjustment of antihyperglycemic therapy needs to be carried out carefully to prevent hypoglycemia, especially in the case of very-low-calorie diets [28].

***Patients with lack of BS nutritional knowledge.*** How, and in what way preoperative programs are offered may influence patients’ ability to process information [29]. A systematic review of patient education practices in BS reported that preoperative educational programs lack standardization, and variation exists among centers in terms of curriculum, timeline, and pathway of delivery [29]. Findings from several published studies suggest that the level of nutritional knowledge among patients seeking to undergo BS might be relatively low [30,31]. Therefore, it seems that interventions aimed at improving the necessary presurgical nutrition knowledge are important [8], even though the impact of a lack of pre-surgical nutrition knowledge on long-term results are currently unclear [30]. A non-RCT of 200 pre-BS patients recruited while attending a pre-BS committee tested an intervention in the form of a 15 min online lecture implemented 1–2 weeks prior to surgery, and found a significantly higher improvement in nutrition knowledge post-intervention within the intervention group [30]. Other study tested the effect of nutrition educational intervention on BS nutrition knowledge among 153 pre-BS patients, and found an increase in nutrition knowledge from the pre- to the early post-surgery period, but a control group was not included [31]. Another small study among a single group of 20 pre-BS patients used a mobile technology, including encouraging messages and video-based education modules, as a modality to prepare patients for BS, and found positive trends of behavior changes and weight outcomes preoperatively [32]. Ultimately, future intervention educational studies should focus on patients with a BS nutrition knowledge deficit to test this potential at-risk population, although it is difficult to define a lack of appropriate BS nutrition knowledge and there is yet no gold-standard tool to assess it [8]. 

***Clinical implications and future directions.*** The preoperative period is an important time window in which patients should undergo nutritional assessment, preparation, and education; however, at this stage, more well-designed interventional studies are required in order to establish uniform, evidence-based nutritional practice guidelines [8]. The development and testing of nutritional interventions seems extremely crucial when focusing on specific at-risk groups for postoperative nutritional and metabolic complications [8,9]. Finally, in addition to the prominent groups reviewed above, further attention should be paid to other at-risk populations mentioned earlier (e.g., candidates for a revisional BS, childbearing-age women seeking future pregnancy, kidney diseases patients), that to date have been less studied with regards to preoperative nutritional intervention strategies aimed at decreasing postoperative complications [8,9]. These groups require further research and may become a key focus of future rigorous interventional studies testing multidomain and nutritional interventions during the preoperative period.

## Data Availability

No new data were created or analyzed in this study. Data sharing is not applicable to this article.

## Abbreviations

BMI—Body mass index; BS—Bariatric surgery; HbA1c—Hemoglobin A1c; RCT—Randomized controlled trial; T2DM—Type 2 diabetes mellitus.
